# Fast Mechanically Driven Daughter Cell Separation Is Widespread in *Actinobacteria*

**DOI:** 10.1128/mBio.00952-16

**Published:** 2016-08-30

**Authors:** Xiaoxue Zhou, David K. Halladin, Julie A. Theriot

**Affiliations:** a Department of Biochemistry, Stanford University School of Medicine, Stanford, California, USA; b Department of Microbiology and Immunology, Stanford University School of Medicine, Stanford, California, USA; c Howard Hughes Medical Institute, Stanford University School of Medicine, Stanford, California, USA

## Abstract

Dividing cells of the coccoid Gram-positive bacterium *Staphylococcus aureus* undergo extremely rapid (millisecond) daughter cell separation (DCS) driven by mechanical crack propagation, a strategy that is very distinct from the gradual, enzymatically driven cell wall remodeling process that has been well described in several rod-shaped model bacteria. To determine if other bacteria, especially those in the same phylum (*Firmicutes*) or with similar coccoid shapes as *S. aureus*, might use a similar mechanically driven strategy for DCS, we used high-resolution video microscopy to examine cytokinesis in a phylogenetically wide range of species with various cell shapes and sizes. We found that fast mechanically driven DCS is rather rare in the *Firmicutes* (low G+C Gram positives), observed only in *Staphylococcus* and its closest coccoid relatives in the *Macrococcus* genus, and we did not observe this division strategy among the Gram-negative *Proteobacteria*. In contrast, several members of the high-G+C Gram-positive phylum *Actinobacteria* (*Micrococcus luteus*, *Brachybacterium faecium*, *Corynebacterium glutamicum*, and *Mycobacterium smegmatis*) with diverse shapes ranging from coccoid to rod all undergo fast mechanical DCS during cell division. Most intriguingly, similar fast mechanical DCS was also observed during the sporulation of the actinobacterium *Streptomyces venezuelae*.

## IMPORTANCE

Much of our knowledge on bacterial cytokinesis comes from studying rod-shaped model organisms such as *Escherichia coli* and *Bacillus subtilis*. Less is known about variations in this process among different bacterial species. While cell division in many bacteria has been characterized to some extent genetically or biochemically, few species have been examined using video microscopy to uncover the kinetics of cytokinesis and daughter cell separation (DCS). In this work, we found that fast (millisecond) DCS is exhibited by species in two independent clades of Gram-positive bacteria and is particularly prevalent among the *Actinobacteria*, a diverse group that includes significant pathogens as well as bacteria that generate medically important antibiotics.

## Observation

The final step of bacterial cell division, daughter cell separation (DCS), is typically a slow process requiring several minutes. In many well-characterized bacteria, including *Escherichia coli* and *Caulobacter crescentus*, DCS is achieved by gradual symmetric constriction coupled with construction of new hemispherical poles at the junction between the presumptive daughters (1, 2), while other bacteria such as *Bacillus subtilis* initially build a flat septum that then undergoes gradual resolution around the periphery to allow symmetric DCS (3). In contrast, the Gram-positive coccus *Staphylococcus aureus* undergoes rapid (millisecond time scale) DCS (4, 5), and the resulting daughters remain connected asymmetrically by a hinge, hallmarks of separation driven by mechanical rupture rather than by gradual enzymatic remodeling of the peripheral cell wall (4).

**FIG 1:**
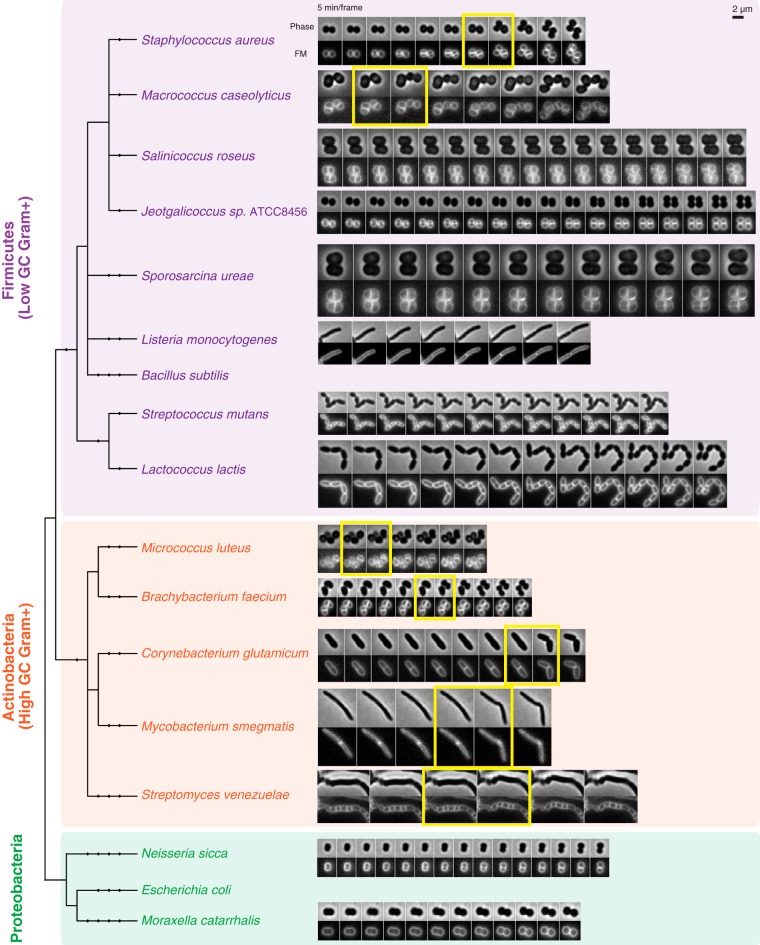
Time-lapse microscopy of DCS for phylogenetically distinct bacteria. Bacterial cells were stained with the membrane dye FM 4-64 and imaged on agarose pads at 5-min intervals. Fast DCS events (the first one for each montage) are highlighted with the yellow boxes. All cells are shown at the same magnification (scale bar, 2 µm). The phylogenetic tree was generated with phyloT based on the NCBI taxonomy and visualized with iTOL (23).

**FIG 2:**
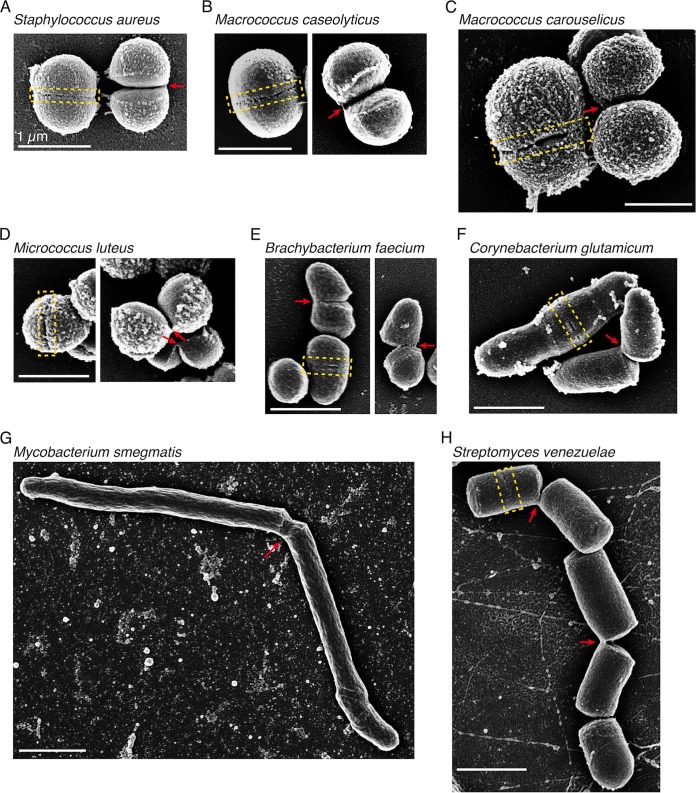
SEM of bacteria that undergo fast DCS. Shown are representative SEM images of “snapping-positive” species. Yellow boxes highlight the surface perforations formed at the peripheral ring prior to DCS, and red arrows highlight the hinges that connect the asymmetrically arranged daughters after DCS. All scale bars are 1 µm.

In order to determine whether this mechanism of fast mechanical DCS is unique to *S. aureus* or also found among other bacterial species, we surveyed representative species across three major bacterial phyla, including the *Firmicutes* (low G+C Gram positives), *Actinobacteria* (high G+C Gram positives), and *Proteobacteria* (Gram negatives), with particular attention to include diverse species that share the coccoid (near-spherical) shape of *S. aureus* (6) (Fig. 1; see Table S1 in the supplemental material). For all species, we directly examined their cytokinesis and DCS processes using time-lapse microscopy, observing both changes in overall cell shape with phase-contrast imaging and reorganization of cell membrane using the intercalating dye FM 4-64 (Fig. 1). Where initial time-lapse characterization using 5-min imaging intervals indicated the possibility of fast mechanical DCS, we further examined cell division using high-speed phase-contrast imaging at 10-ms intervals (see Fig. S1 and Movie S1 in the supplemental material) and scanning electron microscopy (SEM) to characterize the shapes and surface characteristics of cells immediately before and after DCS (Fig. 2).

We first set out to determine whether close relatives of *S. aureus* in the *Staphylococcaceae* family employ fast mechanical DCS. Indeed, *Macrococcus caseolyticus*, which has a similar cell shape but slightly larger size (7), divided like *S. aureus*, such that the round cell gradually formed a septum generating two “hemispherical” daughters which then separated rapidly (within 10 ms) accompanied by a drastic shape conversion (Fig. 1; see Fig. S1B in the supplemental material), resulting in asymmetrically hinged sister pairs (Fig. 2B). Similar behaviors were observed for all four *Macrococcus* species examined (Fig. 2C; see Table S1 in the supplemental material). Surprisingly, two other coccoid members of the *Staphylococcaceae*, *Salinicoccus roseus* (a halophile that grows optimally with 10% salt [8]) and *Jeotgalicoccus* sp. strain ATCC 8456 (a member of a genus originally isolated from the Korean fish sauce jeotgal [9]), showed no evidence of fast mechanical DCS and instead separated by gradual and symmetrical resolution of the septum (Fig. 1; see Fig. S2A and B in the supplemental material). Notably, *S. roseus* formed regular, symmetrical cuboidal clusters (Fig. 1; see Fig. S2A) rather than the irregular “grape-like” clusters characteristic of *S. aureus*, consistent with the idea that irregular clusters are likely to be a consequence of the randomly positioned asymmetric hinge attachment generated by fast mechanically driven DCS (4) while cuboidal clusters of coccoid bacteria may reflect slow and symmetric DCS events.

To compare the behavior of these coccoid *Staphylococcaceae* to related species in the same order, *Bacillales*, we next examined *Sporosarcina ureae*, a large coccoid soil bacterium, and *Listeria monocytogenes*, a rod-shaped pathogen. Both species separated by gradual resolution of the septum (Fig. 1). Additionally, *S. ureae* formed cuboidal clusters similar to *Salinicoccus roseus* (Fig. 1; see Fig. S2C), consistent with symmetric DCS. In addition to the *Bacillales*, we inspected *Streptococcus mutans* and *Lactococcus lactis*, two members of a related order, *Lactobacillales*, both of which have ovoid shapes that divide in a single plane to form chains. Cytokinesis in *S. mutans* and *L. lactis* appeared very similar, where a septum was formed and resolved gradually to form the new poles (Fig. 1), similar to *B. subtilis*. Thus, the closely related genera *Staphylococcus* and *Macrococcus* are the only examples we found of fast mechanically driven DCS among the *Firmicutes*, and this particular behavior was not even observed among all *Staphylococcaceae*.

To explore beyond *Firmicutes*, we next examined two coccoid Gram-negative species among the *Proteobacteria* with different cell sizes: the betaproteobacterium *Neisseria sicca* and the gammaproteobacterium *Moraxella catarrhalis*. Both *N. sicca* and *M. catarrhalis* constricted gradually at the division site to form the new poles while separating the daughters (Fig. 1). This is consistent with the cytokinesis process well documented in rod-shaped Gram-negative bacteria, where DCS coincides with septation to coordinate outer membrane synthesis (1).

Next we turned to the other major Gram-positive phylum besides the *Firmicutes*, the high-G+C *Actinobacteria*. Again we began with a well-characterized coccoid species, *Micrococcus luteus*, the type strain of the genus *Micrococcus* within the *Actinomycetales* (10) known for the discovery of lysozyme (11). Similar to *S. aureus*, daughter cells of *M. luteus* separated rapidly (slower than *S. aureus*, but still within a few tens of milliseconds) (see Fig. S1C in the supplemental material), leaving behind clearly hinged sister pairs (Fig. 2D) and irregular clusters as a result. Similar fast DCS was also observed in *Brachybacterium faecium*, another member in the *Micrococcineae* suborder with a slightly elongated cell shape (12) (Fig. 2E; see Fig. S1D).

One well-known suborder in *Actinobacteria* is the mycolate-producing *Corynebacterineae*, which contains the genera *Corynebacterium* and *Mycobacterium*, both polar-growing rods that have been reported to undergo drastic “V-snapping” at the final step of cell division (13–15). Indeed, we observed that *C. glutamicum* and *M. smegmatis* snapped rapidly following septation, with a characteristic DCS time of ~10 ms (Fig. S1E and F), very similar to the mechanically driven DCS described above for the various coccoid species. Because these organisms are rod shaped, the newly separated daughters connected by a hinge point had an overall V shape as previously described (13–15) (Fig. 2F and G).

For *M. smegmatis*, besides the characteristic V-snapping, we observed another more subtle form of separation where the two daughters remained aligned and symmetric postseparation (labeled “straight” in Fig. S3A to D in the supplemental material), resembling the straight cell form previously reported for *Mycobacterium* cultures (14). However, unlike the gradual symmetric DCS observed in the *Firmicutes* such as *Listeria*, the straight mode of DCS in *M. smegmatis* occurred rapidly with a time scale comparable to that of the V-snapping (within 20 ms; see Fig. S3B), suggesting a similar mechanical mechanism. Given the thin rod shape of *M. smegmatis* (lowest pole size/cell length ratio among all of the species undergoing fast DCS), we wondered whether the fast straight DCS could rise from a scenario in which the torque generated during the asymmetric fracture of the peripheral ring is not strong enough to overcome the resistance for the daughters to rotate around the hinge. Indeed, factors that increase the rotation resistance, such as physical confinements (see Fig. S3E) and adhesions between daughters at the septum presumably due to the mycomembrane (see Fig. S3F), did raise the likelihood of straight DCS.

Finally, we looked at *Streptomyces*, the largest genus in *Actinobacteria* with a complex life cycle, including a vegetative growth stage that yields multigenomic hyphae (substrate mycelia) and a later sporulation stage in which the aerial hyphae septate into spores, typically in response to unfavorable conditions (16). We imaged the sporulation of *Streptomyces venezuelae* hyphae (17) by exposing them to the spent media of a sporulated culture either in microfluidic chambers (see Movie S2 and Fig. S4 in the supplemental material) or on agarose pads (Fig. 1) and observed that separation of the spores is fast and hinged, similar to the “V-snapping” observed in other *Actinobacteria*. Interestingly, we often observed a “chain reaction” process where several parts on the same hypha would snap simultaneously or in rapid succession, possibly due to the buildup of tension in the hypha as a result of adjacent cells snapping (see Movie S2 and Fig. S4). Asymmetric hinge point connections between neighboring spores in a single hyphal chain were readily observable by SEM (Fig. 2H). Thus, so far, all five species representing five distinct families in *Actinobacteria* that we examined undergo fast DCS.

Taken together, our results indicate that cell shape (coccoid, rod, or hyphal) is not a determining factor for whether a particular bacterial species can undergo fast mechanical DCS, while a thick layer of peptidoglycan (Gram positive) together with the formation of a flat septum may be prerequisites. The species we identified here as sharing this feature represent a substantial phylogenetic diversity, yet the mechanisms they use are likely very similar to that of *S. aureus*, with the key factor being the septum structure, where the two daughter cells are predominantly only connected by the peripheral ring postseptation (see Fig. S5 in the supplemental material). Transmission electron microscopy (TEM) images of several *Actinobacteria* species confirmed this septum geometry (15, 18–22). It is intriguing that fast mechanically driven DCS is narrowly distributed in *Firmicutes*, observed in only *Staphylococcus* and *Macrococcus*, while widely adopted in the distantly related *Actinobacteria*. Overall, our findings revealed that the mechanical rupture of the peripheral cell wall is a common strategy implemented by diverse Gram-positive bacteria to accomplish DCS.

### Methods. (i) Bacterial strains and growth conditions.

The strains and corresponding growth conditions are summarized in Table S1 in the supplemental material. For all experiments, overnight cultures were diluted 1:100 into fresh medium and grown until the mid-exponential phase. Live cell imaging was performed on 1% agarose pads prepared with fresh media or in CellASIC B04A plates (EMD Millipore, Inc.). One microgram/ml FM 4-64 (Life Technologies) was added to cultures or agarose pads when needed to stain the cell membrane for time-lapse microscopy.

### (ii) Light microscopy.

Two-dimensional (2D) time-lapse imaging was performed on a Nikon Eclipse Ti inverted fluorescence microscope with a 100× (NA 1.40) oil-immersion objective (Nikon Instruments) and MicroManager v1.4. Cells grown on agarose pads were maintained at the targeted temperature during imaging with an active-control environmental chamber (Haison Technology). An iXon3 888 electron-multiplying charge-coupled device (EMCCD) camera (Andor) was used for fluorescent time-lapse microscopy experiments, and a Zyla 5.5 sCMOS camera (Andor) was used for millisecond phase-contrast imaging of cell separation.

### (iii) Scanning electron microscopy.

Bacterial cells (mid-log phase) were pelleted and resuspended in cold phosphate-buffered saline (PBS) and were fixed with 2% glutaraldehyde and 4% paraformaldehyde in 0.1 M sodium cacodylate buffer (pH 7.3) at 4°C overnight. Fixed cells were settled onto poly-l-lysine (Sigma-Aldrich)-treated coverslips for 2 min on ice and washed with 0.1 M sodium cacodylate buffer three times, postfixed with 1% OsO_4_ at 4°C for 1 h, dehydrated in a series of increasing concentrations of ethanol (50, 70, 95, and 100%), and inserted into an Autosamdri-815 series A critical point dryer (Tousimis) to remove residual ethanol with carbon dioxide. The dehydrated samples were then sputter coated with gold-palladium to an ~60 Å thickness and visualized with a Sigma series field emission scanning electron microscope (Zeiss).

## SUPPLEMENTAL MATERIAL

Figure S1  High-speed phase-contrast imaging of bacteria undergoing fast DCS. Representative montages of DCS captured with 10-ms intervals. White arrows indicate the cells that are about to separate. All scale bars are 2 µm. See also Movie S1 in the supplemental material. Download Figure S1, PDF file, 2.1 MB

Figure S2  SEM of *Bacillales* that undergo slow DCS. SEM images of *S. roseus* (A), *Jeotgalicoccus* (B), *S. ureae* (C), *L. monocytogenes* (D), and *B. subtilis* (E) show intermediate stages of the gradual and symmetric DCS process. Yellow boxes highlight surface perforations formed at the peripheral ring prior to DCS similar to those of the species that undergo fast DCS (Fig. 2). The difference is that even after those perforations grow and merge to dissolve the boundary, the two daughter cells are still connected by the remaining portion of the septum and align parallel to each other symmetrical to the septum. Scale bars represent 1 µm. Download Figure S2, PDF file, 2.8 MB

Figure S3  Two modes of DCS for *M. smegmatis*. (A and B) Examples of the “V-shaped” (top) and “straight” (bottom) modes of DCS in *M. smegmatis* captured at 1 min per frame (A) and 10 ms per frame (B). (C) Representative lineage of *M. smegmatis* where the mother cell undergoes “V-shaped” separation (arrow 1) and one of the daughters undergoes “straight” mode separation (arrow 3). (D) SEM images of *M. smegmatis* grown in a shaking broth culture display the “straight” mode. Scale bars represent 2 µm. (E) Fraction of the “straight” mode separation for cells grown under various degrees of confinement in *z* (left, different experimental conditions) and *xy* (right, number of sides with neighbors). For confinement in *z*, 53 separation events from 10 fields were recorded for cells grown on an agarose pad (agar pad), 328 separation events from two separate experiments with 4 fields each were collected for cells grown in the relatively thick regions of CellASIC chambers (loose trap, where cells were not trapped completely), and 216 division events from two separate experiments with 4 fields each were collected for cells grown in the relatively thin regions of CellASIC chambers (tight trap, where cells were trapped tightly). For confinement in *xy*, separation events of cells grown in CellASIC chambers were categorized based on the number of sides with neighbors. (F) Fraction of straight DCS observed for cells growing in 7H9 with or without adding 0.05% Tween 80, which helps disperse cell clustering through reduction of adhesion between cells. Results recorded from all division events (left) and division events of only single cells without neighbors (right) show the same trend between conditions. Download Figure S3, PDF file, 2.4 MB

Figure S4  Fast DCS in sporulating *Streptomyces venezuelae*. Snapshots of DCS during sporulation of *S. venezuelae* were recorded by phase-contrast microscopy at 5-min intervals. Sporulation was induced by applying spent medium of a sporulated culture to hyphae grown in a microfluidic chamber. The scale bar represents 2 µm. See also Movie S2 in the supplemental material. Download Figure S4, PDF file, 0.6 MB

Figure S5  Three modes of cytokinesis in bacteria. The three major approaches employed by different types of bacteria examined in this study to accomplish DCS are illustrated. Only the center region of the cell with the simplified cell envelope and cytoplasm are shown. The new cell envelope made during cytokinesis, the septum/cross-wall that eventually constitutes the new poles, is drawn in gray to distinguish it from the previous peripheral cell envelope (black). The peripheral ring is indicated in green, while the “glue” material connecting the two septal plates is shown in lighter gray. The glue in Gram-positive species that undergo slow DCS likely involves unresolved peptidoglycan that requires additional enzymatic activities to separate, is much stronger (indicated with darker gray), and serves as the major constraint to hold the two daughters together. In contrast, the two daughters in the Gram-positive species that undergo fast DCS are predominately only connected by the peripheral ring. DCS in both Gram-positive species starts with perforations formed in the peripheral ring. In Gram-positive species with slow DCS, after the peripheral ring is resolved, the two daughter cells separate gradually (minutes) and symmetrically through enzymatic activities that resolve the “glue.” In species with fast DCS, the perforations, once having reached a critical point, initiate the fast (milliseconds) mechanical final separation that resolves the peripheral ring asymmetrically, leaving the two daughters connected by a hinge point in most cases. The scars that originated from the previous peripheral ring material are marked on the new daughters’ surface. Download Figure S5, PDF file, 0.5 MB

Movie S1  Fast DCS captured at 100 frames per second. Representative examples of the fast DCS process were recorded by phase-contrast microscopy with 10-ms intervals for species identified in Fig. 1. All scale bars are 2 µm. See also Fig. S1 in the supplemental material. Download Movie S1, MOV file, 1.0 MB

Movie S2  Sporulation of *Streptomyces venezuelae*. To capture the growth and sporulation of *S. venezuelae*, spores of *S. venezuelae* were loaded into a microfluidic chamber. Fresh medium was first supplied to the chamber for 6 h to induce germination and hyphae growth (vegetative growth), and then spent medium of an already sporulated culture was applied to induce sporulation. This process was recorded by phase-contrast microscopy at 5-min intervals. The scale bar represents 2 µm. See also Fig. S4 in the supplemental material. Download Movie S2, AVI file, 3.2 MB

Table S1  Strains and growth conditions used in this study. Table S1, PDF file, 0.1 MB
