# Supplementary material for: Fast Mechanically Driven Daughter Cell Separation Is Widespread in Actinobacteria
Source: mBio. 2016 Aug 30;7(4):e00952-16. doi: 10.1128/mBio.00952-16 (PMC4999543; doi:10.1128/mBio.00952-16)
Supplement: Figure S1 — High-speed phase-contrast imaging of bacteria undergoing fast DCS. Representative montages of DCS captured with 10-ms intervals. White arrows indicate the cells that are about to separate. All scale bars are 2 µm. See also Movie S1 in the supplemental material. Download [file mbo004162956sf1.pdf]

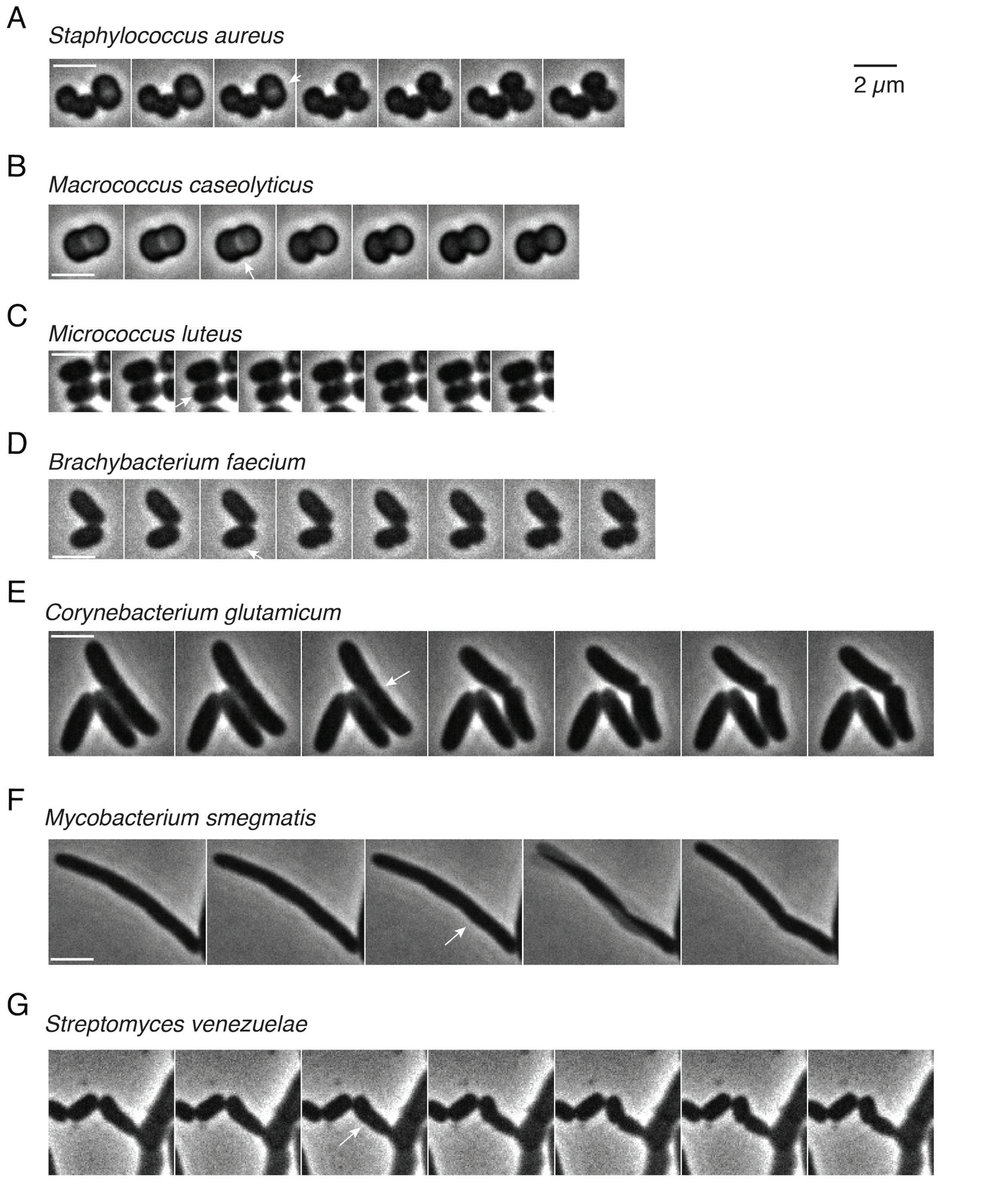
A
Staphylococcus aureus
2 μm
B
Macrococcus caseolyticus
C
Micrococcus luteus
D
Brachybacterium faecium
E
Corynebacterium glutamicum
F
Mycobacterium smegmatis
G
Streptomyces venezuelae
