# Supplementary material for: Fast Mechanically Driven Daughter Cell Separation Is Widespread in Actinobacteria
Source: mBio. 2016 Aug 30;7(4):e00952-16. doi: 10.1128/mBio.00952-16 (PMC4999543; doi:10.1128/mBio.00952-16)
Supplement: Figure S2 — SEM of Bacillales that undergo slow DCS. SEM images of S. roseus (A), Jeotgalicoccus (B), S. ureae (C), L. monocytogenes (D), and B. subtilis (E) show intermediate stages of the gradual and symmetric DCS process. Yellow boxes highlight surface perforations formed at the peripheral ring prior to DCS similar to those of the species that undergo fast DCS (Fig. 2). The difference is that even after those perforations grow and merge to dissolve the boundary, the two daughter cells are still connected by the remaining portion of the septum and align parallel to each other symmetrical to the septum. Scale bars represent 1 µm. Download [file mbo004162956sf2.pdf]

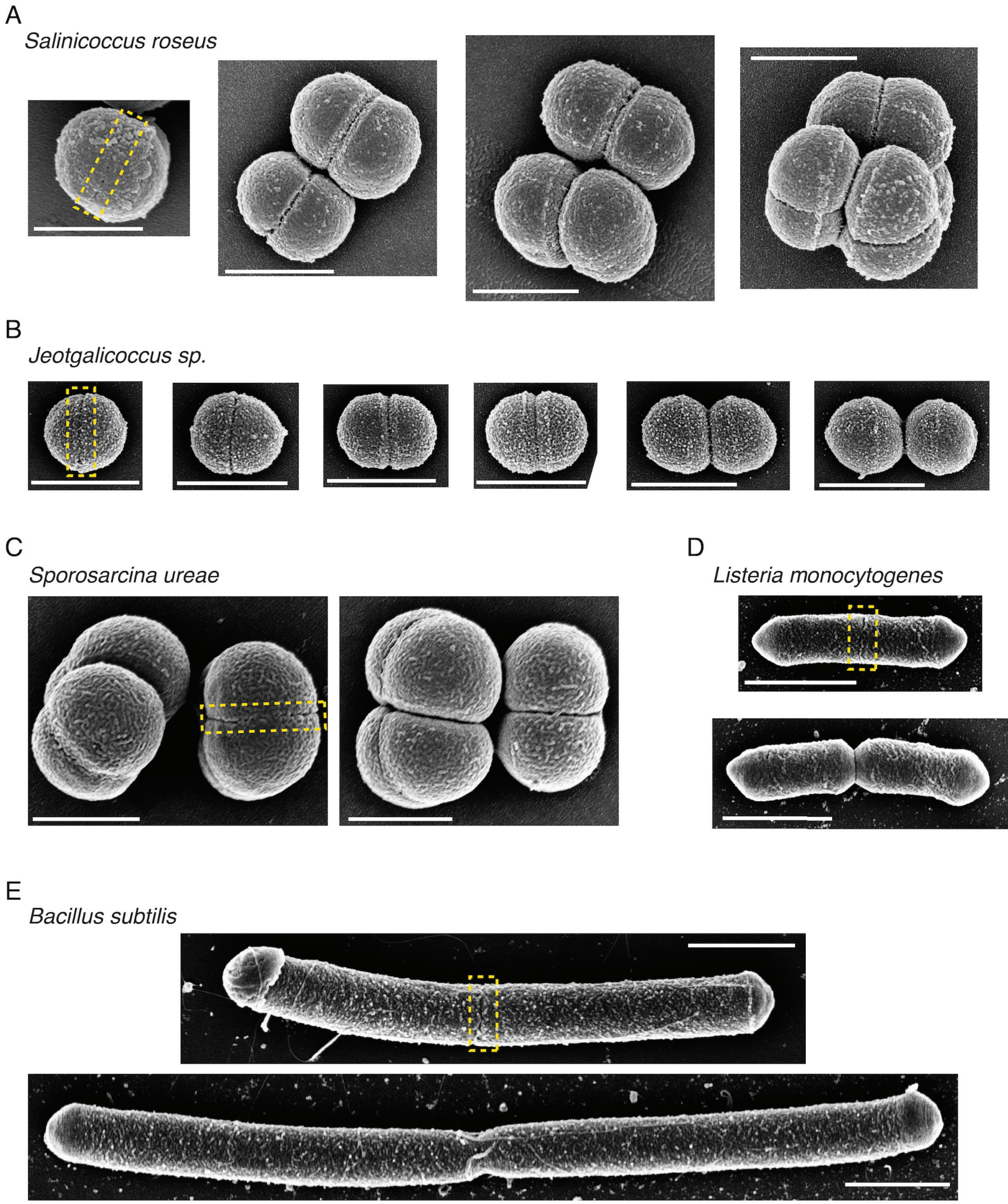

A
Salinicoccus roseus
B
Jeotgalicoccus sp.
C
Sporosarcina ureae
D
Listeria monocytogenes
E
Bacillus subtilis
