# Supplementary figures and images for: Fast Mechanically Driven Daughter Cell Separation Is Widespread in Actinobacteria
Source: mBio. 2016 Aug 30;7(4):e00952-16. doi: 10.1128/mBio.00952-16 (PMC4999543; doi:10.1128/mBio.00952-16)

A

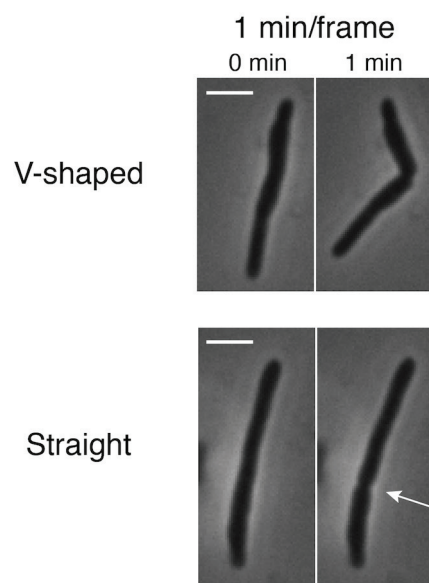

B

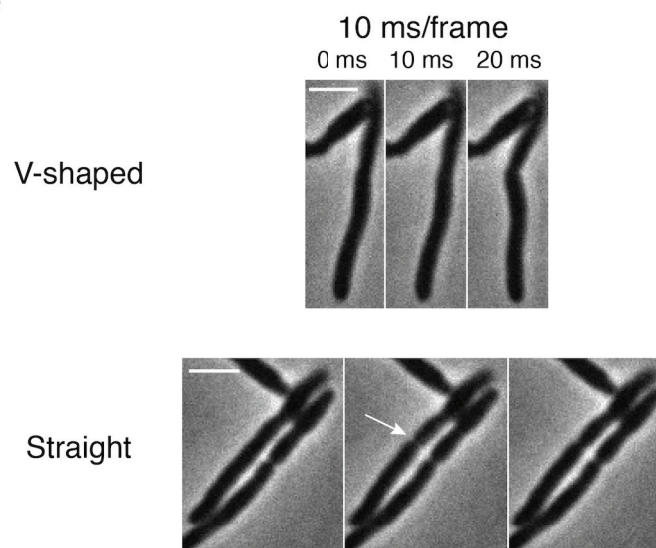

C

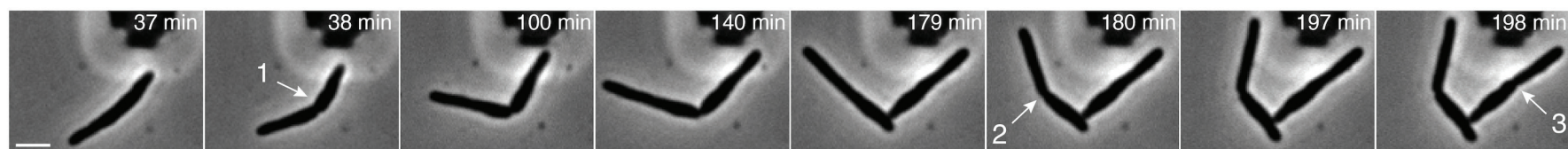

D

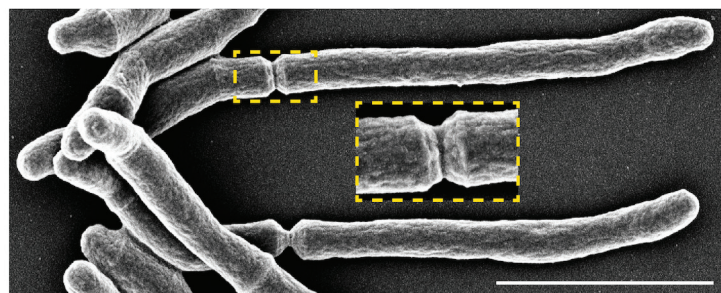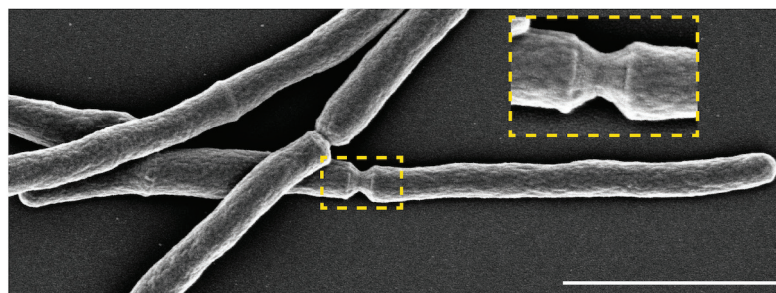

E

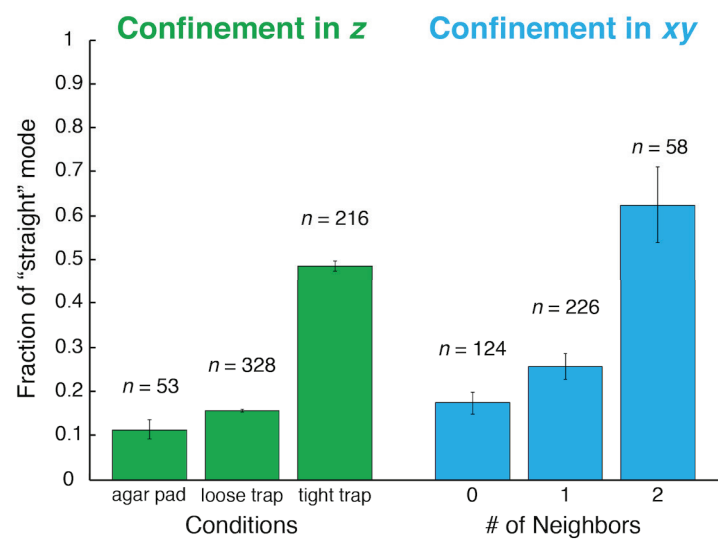

F

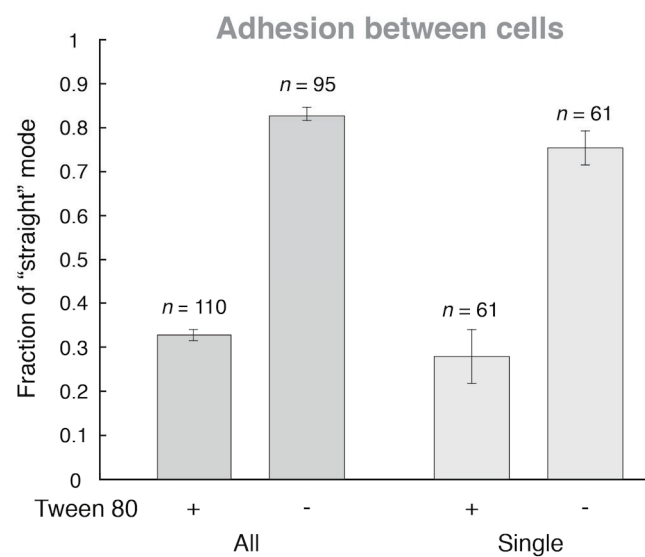

Supplement: Figure S3 — Two modes of DCS for M. smegmatis. (A and B) Examples of the “V-shaped” (top) and “straight” (bottom) modes of DCS in M. smegmatis captured at 1 min per frame (A) and 10 ms per frame (B). (C) Representative lineage of M. smegmatis where the mother cell undergoes “V-shaped” separation (arrow 1) and one of the daughters undergoes “straight” mode separation (arrow 3). (D) SEM images of M. smegmatis grown in a shaking broth culture display the “straight” mode. Scale bars represent 2 µm. (E) Fraction of the “straight” mode separation for cells grown under various degrees of confinement in z (left, different experimental conditions) and xy (right, number of sides with neighbors). For confinement in z, 53 separation events from 10 fields were recorded for cells grown on an agarose pad (agar pad), 328 separation events from two separate experiments with 4 fields each were collected for cells grown in the relatively thick regions of CellASIC chambers (loose trap, where cells were not trapped completely), and 216 division events from two separate experiments with 4 fields each were collected for cells grown in the relatively thin regions of CellASIC chambers (tight trap, where cells were trapped tightly). For confinement in xy, separation events of cells grown in CellASIC chambers were categorized based on the number of sides with neighbors. (F) Fraction of straight DCS observed for cells growing in 7H9 with or without adding 0.05% Tween 80, which helps disperse cell clustering through reduction of adhesion between cells. Results recorded from all division events (left) and division events of only single cells without neighbors (right) show the same trend between conditions. Download [file mbo004162956sf3.pdf]

710 min

715 min

720 min

725 min

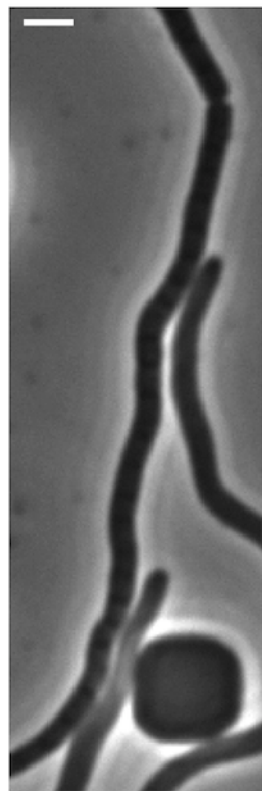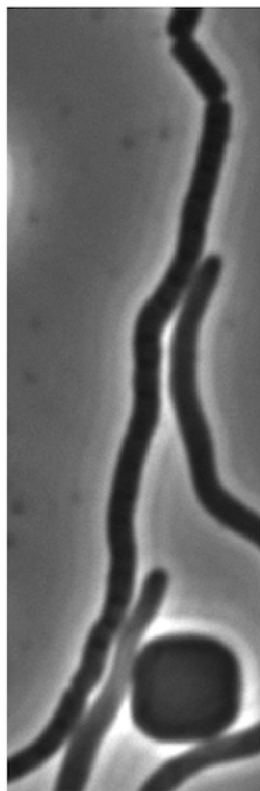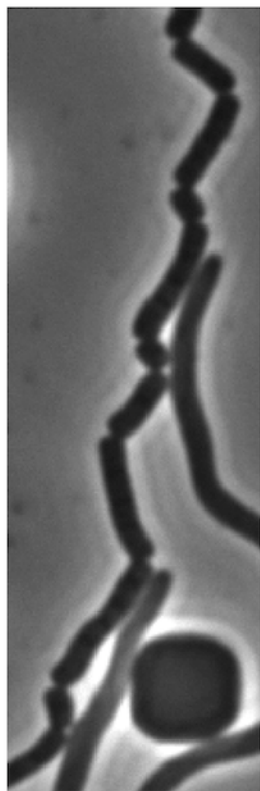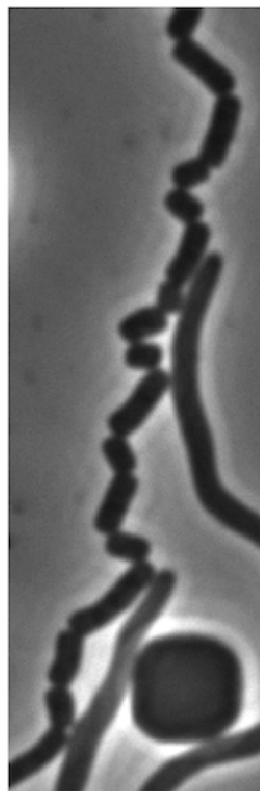

Supplement: Figure S4 — Fast DCS in sporulating Streptomyces venezuelae. Snapshots of DCS during sporulation of S. venezuelae were recorded by phase-contrast microscopy at 5-min intervals. Sporulation was induced by applying spent medium of a sporulated culture to hyphae grown in a microfluidic chamber. The scale bar represents 2 µm. See also Movie S2 in the supplemental material. Download [file mbo004162956sf4.pdf]

# Gram-negative

# Slow DCS Gram-positive

# Fast DCS Gram-positive

cell envelope —●—  
cytoplasm —●—

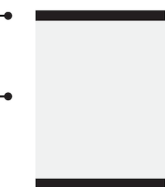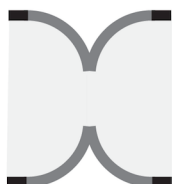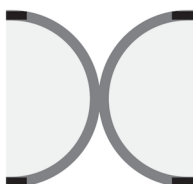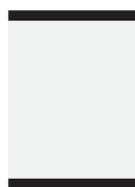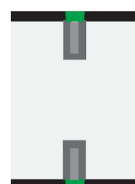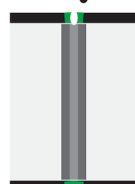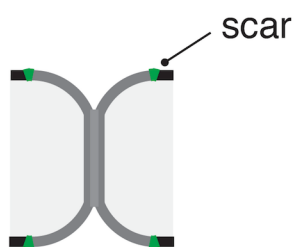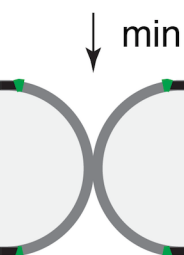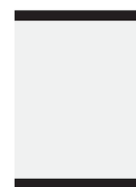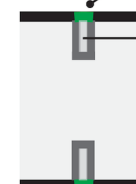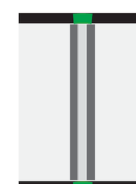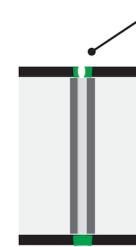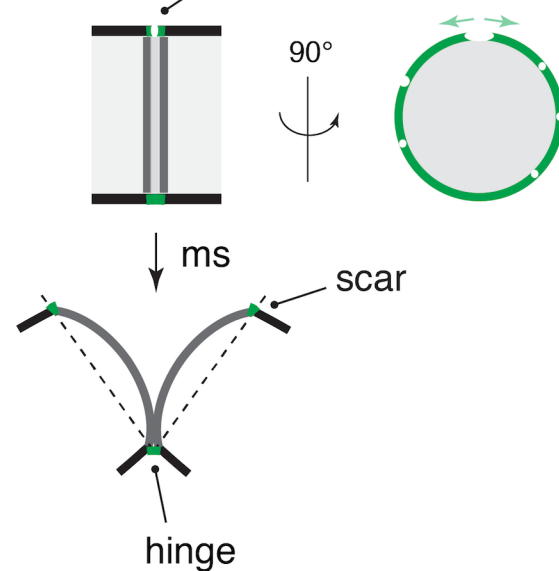

Supplement: Figure S5 — Three modes of cytokinesis in bacteria. The three major approaches employed by different types of bacteria examined in this study to accomplish DCS are illustrated. Only the center region of the cell with the simplified cell envelope and cytoplasm are shown. The new cell envelope made during cytokinesis, the septum/cross-wall that eventually constitutes the new poles, is drawn in gray to distinguish it from the previous peripheral cell envelope (black). The peripheral ring is indicated in green, while the “glue” material connecting the two septal plates is shown in lighter gray. The glue in Gram-positive species that undergo slow DCS likely involves unresolved peptidoglycan that requires additional enzymatic activities to separate, is much stronger (indicated with darker gray), and serves as the major constraint to hold the two daughters together. In contrast, the two daughters in the Gram-positive species that undergo fast DCS are predominately only connected by the peripheral ring. DCS in both Gram-positive species starts with perforations formed in the peripheral ring. In Gram-positive species with slow DCS, after the peripheral ring is resolved, the two daughter cells separate gradually (minutes) and symmetrically through enzymatic activities that resolve the “glue.” In species with fast DCS, the perforations, once having reached a critical point, initiate the fast (milliseconds) mechanical final separation that resolves the peripheral ring asymmetrically, leaving the two daughters connected by a hinge point in most cases. The scars that originated from the previous peripheral ring material are marked on the new daughters’ surface. Download [file mbo004162956sf5.pdf]
