# Supplementary material for: Fast Mechanically Driven Daughter Cell Separation Is Widespread in Actinobacteria
Source: mBio. 2016 Aug 30;7(4):e00952-16. doi: 10.1128/mBio.00952-16 (PMC4999543; doi:10.1128/mBio.00952-16)
Supplement: Table S1 — Strains and growth conditions used in this study. [file mbo004162956st1.pdf]

Table S1. Strains and growth conditions used in this study

Species	Strains	Medium	Temperature	Fast DCS
Staphylococcus aureus	Newman (P. Levin)	TSB	37 ºC	+
Macrococcus caseolyticus	ATCC 13548	TSB	37 ºC	+
Macrococcus equipercicus	ATCC 51831	TSB	37 ºC	+
Macrococcus bovicus	ATCC 51825	TSB	37 ºC	+
Macrococcus carouselicus	ATCC 51828	TSB	37 ºC	+
Salinicoccus roseus	ATCC 49258	1708 HM	30 ºC	-
Jeotgalicoccus sp.	ATCC 8456	TSBY[a]	30 ºC	-
Sporosarcina ureae	ATCC 13881	TSB	26 ºC	-
Listeria monocytogenes	10403S (lab stock)	BHI	37 ºC	-
Bacillis subtilis	168 (lab stock)	LB	37 ºC	-
Streptococcus mutans	ATCC 25175	BHI	37 ºC	-
Lactococcus lactis	ATCC 11454	BHI	37 ºC	-
Neisseria sicca	ATCC 9913	BHI	37 ºC	-
Moraxella catarrhalis	ATCC 8176	BHI	37 ºC	-
Micrococcus luteus	ATCC 4698	TSB	30 ºC	+
Brachybacterium faecium	ATCC 43885	TSB	26 ºC	+
Corynebacterium glutamicum	ATCC 13032	BHI	30 ºC	+
Mycobacterium smegmatis	ATCC 700084	7H9	37 ºC	+
Streptomyces venezuelae	ATCC 10712	ISP1/2	26 ºC	+

[a] TSBY = TSB + 0.5% Yeast extract.
